# Targeting patients for multimorbid care management interventions: the case for equity in high-risk patient identification

**DOI:** 10.1186/1475-9276-12-70

**Published:** 2013-08-20

**Authors:** Efrat Shadmi, Tobias Freund

**Affiliations:** 1 Faculty of Social Welfare and Health Sciences, University of Haifa, Mount Carmel 31905, Israel; 2 Department of General Practice and Health Services Research, University Hospital Heidelberg, Heidelberg, Germany

**Keywords:** Multimorbidity, Equity, High-risk patients, Care management

## Abstract

Targeting patients for multimorbid care management interventions requires accurate and comprehensive assessment of patients’ need in order to direct resources to those who need and can benefit from them the most. Multimorbid patient selection is complicated due to the lack of clear criteria - unlike disease management programs for which patients with a specific condition are identified. This ambiguity can potentially result in inequitable selection, as biases in selection may differentially affect patients from disadvantaged population groups. Patient selection could in principal be performed in three ways: physician referral, patient screening surveys, or by statistical prediction algorithms. This paper discusses equity issues related to each method. We conclude that each method may result in inequitable selection and bias, such as physicians’ attentiveness or familiarity, or prediction models’ reliance on prior resource use, potentially affected by socio-cultural and economic barriers. These biases should be acknowledged and dealt with. We recommend combining patient selection approaches to achieve high care sensitivity, efficiency and equity.

## 

Two major challenges are at play in the design of current and future health care delivery: the growing diversity of patient populations and the increasing burden of multiple long term conditions. The majority of persons 65 and older have multiple chronic conditions, and almost half experience functional limitations associated with chronic disease [1]. Multimorbidity (the co-occurrence of conditions within individuals [2,3]), is consistently shown to be associated with the large share of resource consumption in various population groups and health care settings [4], and with greater burden in minority and disadvantaged populations [5].

Providing care for persons with multimorbidity requires a shift from the prevailing single-disease biomedical health care model, characterized by: lack of scientific evidence on treatments for patients with multiple conditions and deficiencies in teamwork, despite essential participation of professionals from various specialties [6]. Patients with multimorbidity report poor physician-patient communication, worse interpersonal treatment, and lack of care coordination [7,8]. To meet these challenges, care management programs are emerging, aiming to provide better self management support and care coordination [9,10].

The ability of multimorbid care management programs to meet the needs of their target population is, at least in part, contingent on the way patients are selected for entering such programs. Inclusion of patient who are “too ill” and due to their overall morbidity might require other types of care (for example, end of life care) or “too healthy” and do not necessarily need intensive care management, will result in inefficiencies and possibly lack of ability to show program effectiveness.

Patient selection – commonly called case finding – could in principal be performed in three ways: i) physician referral, ii) patient screening tools/Health Risk Assessment (HRA), or iii) statistical prediction of future outcomes. Primary care physicians’ referral decisions are influenced by a complex mix of patient, physician, and health care system characteristics, with no standardized approach to referral decision making [11]. While physician referral is the most common patient selection method, the lack of standardization can lead to inefficiencies in the way patients are targeted for inclusion in resource intensive programs. For example, in a “Get with the Guidelines” study, referral of patients to a heart failure disease management program was examined [12]. The study showed that referral was associated with atrial fibrillation, implanted cardiac device, depression, and treatment at larger hospitals. Yet, paradoxically, patients at higher 90-day mortality risk were less likely to be referred.

Patient screening tools provide a standardized approach for patient selection, based on the fulfillment of specific criteria. An additional virtue of screening tools is that they enable to incorporate information on patient characteristics with administrative and cost data to assess overall risk. Information collected via screening surveys can elicit information which may not be straightforwardly evident to the physician, whose referral is based mainly on clinical knowledge [13]. Similarly, computerized predictive algorithms incorporate information on various risk factors to provide a standard approach for selecting the highest risk patients [14]. Such automated tools prevent the need for resource intensive survey administration and can provide a-priori risk assessments to large numbers of persons (patients or health plan enrollees).

Patient selection tools are commonly used in clinical practice for directing patients for receipt of appropriate health care services, mostly for single disease or single condition programs. Identification of patients for multimorbidity care management provides additional challenges. First, the types of conditions considered and the way multimorbidity is measured define the target population for the program. Measuring multimoridity using a limited set of conditions inevitably implies that selection is limited to patients with one or more conditions from that list. Whether, for example, mental health conditions and/or low prevalence conditions which might be more common in specific population groups, are considered, depends on these a-priori criteria. A “whole-patient oriented” view of disease is more accurate and equitable than an additive account of several conditions. The greater likelihood of occurrence, severity, and adverse effects of health conditions within certain population groups is compounded even further by multiple illnesses, multiple serious illnesses, and greater likelihood of adverse events from incompatible interventions [15]. Thus, multimorbidity measures that account for the entire spectrum of health conditions, interactions, severity and duration, provide a comprehensive classification of multimorbidity, which goes well beyond the additive approach to stratification according to a predetermined number of chronic conditions [16].

Additionally, multimorbid case finding may be based on a predetermined number and types of chronic conditions either by physician referral and/or screening tools like “Probability of Repeated Admissions” (Pra and Pra Plus) [17] or “Identification of Seniors at Risk” (ISAR) [18]. Alternatively, a number of statistical prediction models (PM), based on diagnostic claims or Electronic Medical Record (EMR) and resource use data, aim to identify patients at highest risk for future high resource consumption [14,19,20]. Research shows that such clinical PM models provide better classification than PM that are based on prior use alone [14,20,21].

Compared with physician referral, PM identify patients at higher risk of healthcare utilization but with lower care sensitivity, i.e. likelihood that preventive care will be successful in these cases. Care sensitivity implies three key dimensions: patients’ *willingness* and *ability* to participate in care management programs that are able to *mitigate* their needs [22]. A review of alternative approaches to account for patients’ ‘impactability’ revealed exclusion of highest-risk cases, identification of care gaps and predicted risk of disenrollment as current trends in case finding [23].

## High-risk case identification and equity

High-risk case identification is intended to tailor resources to patients who need them the most. While horizontal equity aims to achieve equal distribution of resources, vertical equity exists when people with greater needs are provided with greater resources [24]. Thus, targeting patients for inclusion in resource intensive care management programs, may be viewed not only as an efficiency measure, but also as a means to achieve greater vertical equity.

The attainment of equity, however, is contingent on the type of high-risk case identification measure used. Approaches that fail to account for the entire spectrum of morbidity may inaccurately reflect need [15], thus resulting in inequitable selection. Moreover, when bias is introduced into the case identification process, equity might be jeopardized, as detailed below. We consider equity issues related to patient selection via physician referrals, patient screening surveys, and automated PMs.

Physician identification for inclusion in care management interventions follows the rational of any medical referral, in which physicians/providers have the responsibility and prerogative to refer patients to services and treatments, according to need. Physicians have information on the entire health and health related needs of patients, often not captured in clinical or administrative databases [22,25,26]. Physicians are able to account for patients’ care sensitivity thereby assisting resource allocation to patients with highest likelihood to benefit from preventive care. Physician referrals also overcome health literacy and language proficiency barriers that may affect patient selection via survey screening [27]. Moreover, physicians can select patients without substantial history of insurance claims or EMR data. Since PMs are based on available diagnostic and resource use data, physician referral may be more equitable as patients enrolled for a short-term period may provide not enough data to be included in PM. Moreover, accessibility, especially for the use of specialty care and expensive imaging services may be lower in marginalized populations, such as those of low socioeconomic status [28,29], and result in an overall lower PM score. Additionally, PMs that are reliant on pharmacy data may be affected by financial accessibility [30], especially if data is derived from purchasing.

Another danger of PM tools is in the potential for misuse – PMs may impede access to care for most vulnerable patients if using socioeconomic status as proxy for non-compliance and therefore non-selection into intensive care management [23]. Nonetheless, this type of non-selection risk exists in all types of patient selection methods.

Patient selection according to physician referral however, is prone to several types of biases. Standardized approaches like PM and patient screening identify patients based on objective criteria rather than relying on physicians attentiveness [23] or personal attitudes [22]. PM tools may identify gaps in (primary) care on an objective basis [23]. In the case of high risk patients that do not have a usual source of care, or have irregular use of health care services, physicians’ knowledge of patient is poor and the physician may not be able to accurately assess their risk. Additionally, the fact that compared with PM physicians tend to select patients with a prior history of participation in intensified care management programs [25] suggests greater care sensitivity of physician referral but may also indicate an “under the lamp-post” bias (i.e., possible bias towards non selection of those with lower adherence or those with accessibility barriers).

## Conclusion

*Active sel*ection of eligible patients for multimorbidity care management is an equity issue. Each of the different case finding approaches provides opportunities for both increasing and decreasing equity in healthcare. Benefits of objective criteria for case finding in PM for example, may at least partly be outweighed by data inputs needs or ‘inequitable’ modeling algorithms. Conversely, physicians may be prone to select their more engaging patients for inclusion in intensive care management, overlooking patients with complex care needs with a history of lower care engagement which may be due to socio-cultural barriers to care. Therefore, health care providers and health authorities should be aware of equity issues whenever selecting multimorbid patients for tailored care management as any selection method principally comes along with not-selecting others, who may be in need of intensified care services. When non-selected patients are more likely to come from disadvantaged population groups (as demonstrated by the “inverse care” law [31]) equity is jeopardized. Current trends in predictive modeling like the introduction of models which incorporate proxies for care sensitivity may or may not increase equity depending on how proxies like socioeconomic status are used (see above).

To conclude, this overview of equity in multimorbid patient selection approaches suggests that combining different case finding approaches like PM and physician referral may help to overcome the pitfalls of each of the two [22,25]. Although rigorous studies comparing the effect of case finding on equity are lacking to date, first attempts show promising results [10].

## Competing interests

ES is co-editor in chief of the International Journal for Equity in Health (unpaid). TF has no competing interests to declare.

## Authors’ contributions

ES and TF conceptualized the paper, summarized the literature findings and drafted the manuscript. Both authors read and approved the final manuscript.
